# Promoting Secondary Students' Twenty-First Century Skills and STEM Career Interests Through a Crossover Program of STEM and Community Service Education

**DOI:** 10.3389/fpsyg.2022.903252

**Published:** 2022-07-06

**Authors:** Biyun Huang, Morris Siu-Yung Jong, Ronnel B. King, Ching-Sing Chai, Michael Yi-Chao Jiang

**Affiliations:** ^1^Centre for Learning Sciences and Technologies, The Chinese University of Hong Kong, Hong Kong, Hong Kong SAR, China; ^2^Department of Curriculum and Instruction, Faculty of Education, The Chinese University of Hong Kong, Hong Kong, Hong Kong SAR, China; ^3^Centre for the Enhancement of Teaching and Learning, Academic Unit of Human Communication, Development, and Information Sciences, The University of Hong Kong, Hong Kong, Hong Kong SAR, China

**Keywords:** STEM education, community service learning, secondary students, twenty-first century skills, creativity, collaboration, perseverance, career interests

## Abstract

STEM education has been regarded as an important educational initiative for cultivating students' twenty-first century skills. The present work aimed to explore ways to promote students' twenty-first century skills through an integrated STEM-based curriculum. Specifically, we designed and implemented an 8-week crossover program of STEM and community service education. In this program, students learned about STEM domain knowledge and community service issues. They then applied the knowledge to solve authentic problems faced by Hong Kong community-housing residents from disadvantaged groups. A mixed-method approach was employed to evaluate the effectiveness of the program in enhancing students' twenty-first century skills and attitudes, including (i) creative thinking, (ii) collaboration, (iii) perseverance, as well as their (iv) STEM career interests. The research participants were 121 secondary students from a government-subsidized school. The quantitative results showed that the participants' creative thinking, collaboration, and perseverance improved alongside their STEM career interests. These findings were further supported by the data gathered through focus-group interviews. This study provides theoretical and practical insights into the integration of STEM education with community service learning.

## Introduction

STEM education is one of the most prominent educational initiatives in the twenty-first century (Geng et al., 2019; Jong et al., 2021; Lau and Jong, 2022). This initiative has been viewed as a promising pathway for nurturing a nation's future workforce and enhancing economic competitiveness (Honey et al., 2014; Chai et al., 2020b; Reynante et al., 2020). Meanwhile, constructivist learning paradigms have been largely promoted in school education since the past two decades (Luk et al., 2006; Jong et al., 2010; Song et al., 2017; Bower and Jong, 2020). Instead of using conventional didactic teaching approaches, successful STEM education programs usually adopt constructivist student-centered approaches with a strong emphasis on learners' active participation (Ulger, 2018; Wan et al., 2021; Weng et al., 2022). Integrating community service learning (Yorio and Ye, 2012; Chiva-Bartoll et al., 2020) with STEM education, where learners apply what they learn to provide solutions to the problems in their communities, is considered an innovative and constructivist approach (Collins et al., 2020; Marcus et al., 2021).

Despite the promise of synergizing community service learning with STEM education, empirical research on the effectiveness of this integration is scarce (Botelho et al., 2020). To address this shortcoming, we explored the integration of community service learning with STEM education. We evaluated its influence on students' twenty-first century skills and their attitudes, including (i) creative thinking, (ii) collaboration, (iii) perseverance, and their interest in STEM careers (Fullan and Langworthy, 2014; Scherer and Gustafsson, 2015; Maiorca et al., 2021).

Twenty-first-century skills pertain to the knowledge, skills, and attitudes needed by children and the youth to fully engage in and contribute to the development of society (Lin et al., 2021). Binkley et al. (2012) summarized that twenty-first century skills could be classified into four groups, including ways of thinking (e.g., creativity and innovation, critical thinking, problem solving, and learning to learn), ways of working (e.g., communication and collaboration), tools for working (e.g., information and ICT literacy), and living in the world (e.g., citizenship, life and career, and sense of responsibility). Chai et al. (2020a) reviewed frameworks for twenty-first century skills and noted that most of the frameworks emphasized the importance of engaging students in creative thinking, critical thinking, and authentic problem solving. In addition, classroom-based and out-of-classroom learning communities should support students in building communication and collaboration skills in the problem-solving process, which contributes to growth in learning and innovation (Chai et al., 2020b). Tsai et al. (2013) expressed that the essence of education in the twenty-first century has to be epistemologically shifted from reproduction of knowledge to production of new knowledge through creativity and innovation.

In response to the prominent call for the educational shift to cultivating creative, competent, and responsible future citizens (Papadakis, 2016), we launched the integrated STEM-based community service program, and examined its impact on students' twenty-first century skills, such as creative thinking and collaboration. Specifically, we situated our study in the context of Hong Kong junior secondary setting and developed a program wherein students learned about the common living problems encountered by community-housing residents (e.g., the problem of having limited housing spaces). The students collaborated to propose solutions in teams.

Aside from these two critical twenty-first century skills, we also examined the impact of the program on students' perseverance and STEM interest. Perseverance pertains to a proactive attitude toward difficulties and the willingness to make continuous efforts to solve problems (Foster and Schleicher, 2022). Perseverance is one of the main characteristics of creative individuals and an important component of creative capacity (Amabile, 1983; Scherer and Gustafsson, 2015). It is an important variable that links to the quality of students' creative problem-solving (Foster and Schleicher, 2022). Moreover, STEM career interest is another important variable because many studies have shown that there are insufficient numbers of students who want to pursue a STEM career (Karahan et al., 2021; Maiorca et al., 2021). Hence, we also examined whether the crossover program could potentially enhance students' interests in STEM careers.

## Literature Review

### Rationale Behind Integrating STEM Education and Community Service Learning

Integrated STEM is an approach that explores teaching and learning between two or more STEM subjects within an authentic context to build connections between disciplinary knowledge and their applications (Kelley and Knowles, 2016; Nadelson and Seifert, 2017). Fostering connections across the disciplines is more likely to promote students' twenty-first century skills, develop a STEM-capable workforce, and boost interest in STEM (Honey et al., 2014; Lee et al., 2019). English (2019) integrated designing ones' own pair of shoes activity with engineering and science disciplines in a fourth-grade STEM course and found that students' design skills and trouble-shooting strategies improved. Morrin and Liston (2020) integrated visual arts with school space design activities and reported that students' attitudes and creativity skills were positively influenced. When accompanied by teamwork, the integrated approach unlocks untapped potential and could benefit learners' knowledge acquisition, higher order thinking, as well as collaboration skills (Moirano et al., 2020).

Although the integration of STEM with different subjects is promising, there is a need for more empirical endeavors to explore how and what could be done to achieve desired learning outcomes. In recent years, scholars have emphasized that STEM education is not just confined to the cognitive and technical realm (i.e., the systematic application of mathematical and scientific knowledge to develop novel solutions to complex problems) (Gunckel and Tolbert, 2018). Rather, it should also involve the cultivation of values, civic attitudes, empathy, and encourage socially responsible and human-centered solutions (Bielefeldt, 2017; Huang et al., 2022). Therefore, advocates have recently suggested that “arts” should be incorporated into STEM education, which refers to a single arts discipline or an expanded area of the liberal arts and humanities disciplines (Perignat and Katz-Buonincontro, 2019; Quigley et al., 2019). The integration of “arts” offers a number of benefits to students (Bush et al., 2020, p. 693), such as engaging more students who do not identify themselves with STEM and providing meaning for learning about STEM knowledge, through the process of “solving authentic problems to make the world a better place.”

Community service learning can provide an authentic sociocultural context for STEM education. It is a form of experiential education that engages students in human-centered service-learning activities to address community needs and develop students' value and knowledge (Jacoby, 1996; Tijsma et al., 2020). The explicit connections between learning objectives and structured community interactions promote a broad appreciation of the discipline and enhance personal growth, values, and civic attitudes (Bringle and Hatcher, 1996; Salam et al., 2019). Evidence of the positive effects of this learning approach has been reported in several studies. For example, Reed et al. (2015) reported that university students who took community service-learning courses demonstrated a higher likelihood of persistence. Gerholz et al. (2018) found that university students who collaborated with charitable organizations in problem-solving projects improved their self-efficacy and self-concept. Burton and Winter (2021) examined two service-learning courses where university students applied history or psychology knowledge to help community partners, and reported that students strengthened communication and problem-solving skills and that service-learning courses raised students' intentions to pursue a service-focused career. These studies exhibited the potent effects of community service learning (Burton and Winter, 2021). Nevertheless, most of the explorations were in higher education sectors. Though Collins et al. (2020) provided an initial exploration on integrating community service learning with STEM education through summer workshops in high school, our knowledge about how to implement crossover programs in formal K-12 classroom settings is limited.

To extend the scholarship and innovative practices in the field, there is a need to further explore other curricular and pedagogical possibilities. The present work aims to provide secondary school students with authentic learning experiences based on real-life problems faced by the Hong Kong community. In particular, the problem of limited living space was chosen to engage students in human-centered design.

### Toward a Design Model of Integrated STEM-Based Community Service Learning

#### Model of Human-Centered Design Thinking

To facilitate human-centered product or solution design, the Hasso Plattner Institute of Design (2010) proposed a five-stage model, namely empathize, define, ideate, prototype, and test (EDIPT). The model has been widely adopted in STEM learning activities for scaffolding innovative design processes. In the empathize stage, opportunities need to be created for designer(s) to observe and interact with potential users in their lived context to understand users' needs. In the define stage, designer(s) decide the challenge to take on based on what they learned about the users in the previous phase. In the ideation stage, designer(s) brainstorm a range of potential solutions to solve the challenge. In the prototype stage, designer(s) build prototypes to get close to the final solution. In the test stage, designer(s) interact with the potential users after they test the prototype and collect feedback to refine the solution.

The EDIPT model emphasizes the procedure of empathizing with users and iterative design based on user feedback. It does not stipulate other elements necessary for scaffolding innovation, such as the environment and prior skills. It has been used either together with other models to guide the design process (e.g., Da Silva et al., 2020) or independently (e.g., Yalçin and Erden, 2021). Yalçin and Erden (2021) implemented the EDPIT model in a pre-school STEM program and engaged the children in a series of design activities, for example, designing a prototype that would help them climb high. Yalçin and Erden (2021) found that the experimental group gained higher scores in creativity, problem-solving, and persistence after completing the program.

#### Componential Model of Creativity

In the componential model of creativity, Amabile and Pratt (2016) summarized the social and psychological components necessary for an individual or team to produce creative work or solutions. Amabile and Pratt (2016) emphasized that there are three primary elements needed for innovation, including resources in the task domain, skills and processes for combing the recourses in new ways, and the motivation to innovate. They further illustrated the five stages of innovation as: (1) identifying the problems to be solved or goals to be achieved; (2) preparing for a successful process, such as gathering resources, collecting information, and assigning tasks; (3) generating possibilities; (4) evaluating possibilities; and (5) assessing outcomes and making decisions based on results achieved.

Amabile and Pratt's (2016) componential model of creativity has been successfully applied in K-12 contexts, particularly in secondary education. For example, Hong and Song (2020) observed the physics class of a secondary school, analyzed students' critical incidents worksheets, and interviewed student participants to identify the components influencing creativity. They found that teacher support, such as guidance in the inquiry process and positive feedback, and classroom environment are closely associated with creativity in science classes which aligns with Amabile's (1983) proposition and other recent arguments underlining the importance of teachers in STEM education (e.g., Geng et al., 2019; Jong et al., 2021; Lau and Jong, 2022). Ginns et al. (2021) surveyed students across 13 secondary schools in Australia and confirmed the predictions based on the componential model, such as a supportive environment for creativity is related to intrinsic motivation and creative self-efficacy. They further identified that intrinsic motivation is conducive to creative self-efficacy. Sun et al. (2020) investigated the relationship between students' skills in the task domain (e.g., domain knowledge) and scientific creativity, and found that students' domain knowledge positively influenced their creativity. The above studies confirmed that it is essential for schools to provide students with resources and raw materials for solving problems, equip students with domain-specific skills and creative process skills, and establish a supportive classroom environment through positive feedback.

#### Proposing an Integrated STEM-Based Community Service-Learning Model

**Figure 1:**
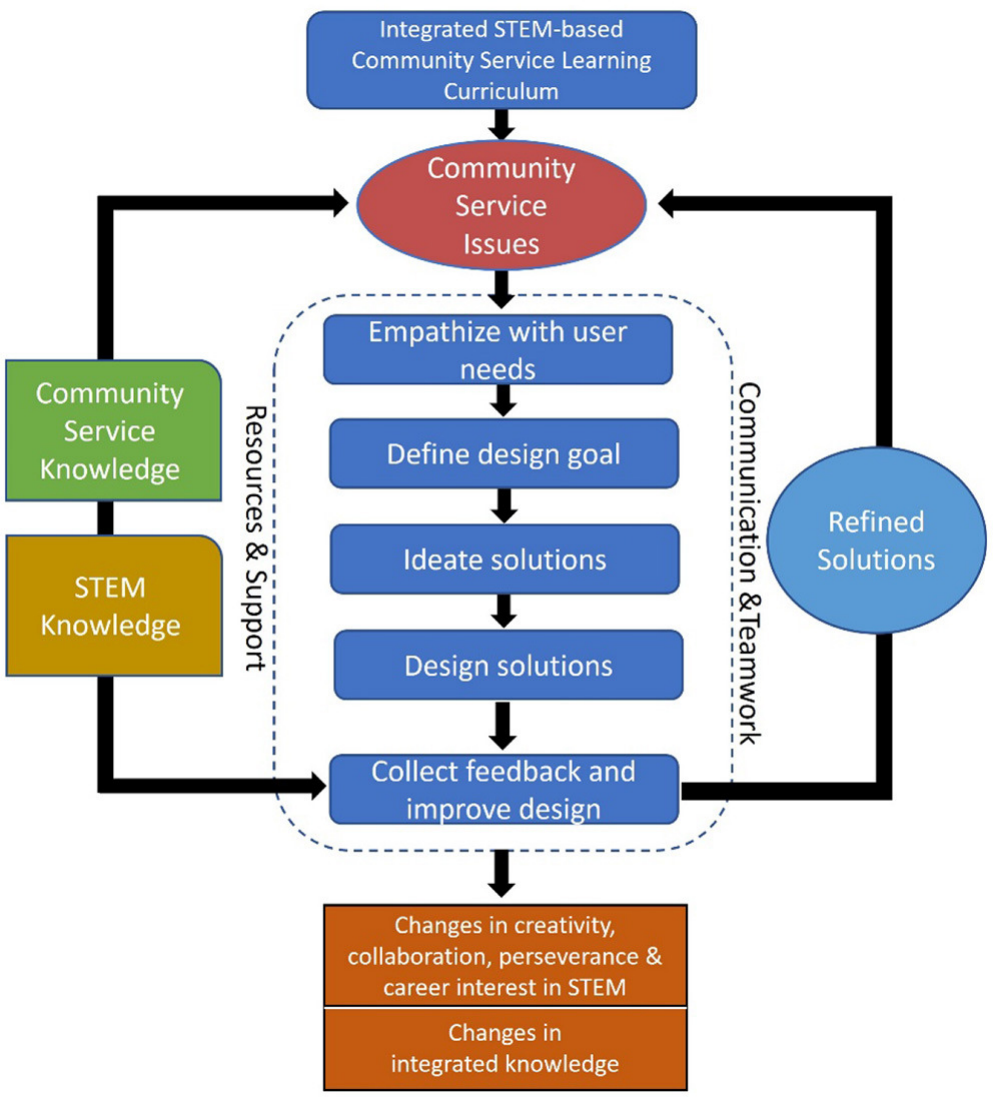
The integrated STEM-based community service-learning model.

Building upon the EDIPT design thinking model and the componential model of creativity, we proposed an integrated STEM-based community service-learning model for designing integrated STEM courses (see Figure 1). The model depicted the main components for supporting individual or team creativity in K-12 setting, such as resources and support from school level, and opportunity to learn relevant domain knowledge. In order to build students' capacity in designing solutions for community service issues, students need to learn foundational knowledge in community service and STEM (Amabile, 1983; Sun et al., 2020). Hence, community service knowledge and STEM knowledge should be included as indispensable parts of the learning model. In the meantime, students should follow a creative process to explore solutions addressing real-world challenges in the community. The exploration can be conducted by individual students or teams, but preferably in team formats, to stimulate better solutions and higher synergy (Mavri et al., 2020). Furthermore, user and expert feedback can be provided to students on improving the designs and creating user-oriented meaningful solutions (Vo and Asojo, 2021). It is assumed that after completing cross-disciplinary curriculums based on the integrated model, students' twenty-first century skills, such as creative thinking, collaboration, perseverance and STEM career interests, would be enhanced.

## Research Design

### Design of the Integrated STEM-Based Community Service Course

In designing the interdisciplinary course, we followed the integrated STEM-based community service-learning model. In the STEM knowledge aspect, students learned concepts and skills on coding, the Internet of things (IoT), smart home devices, and the development of mini devices. In the community service knowledge aspect, students learned foundation knowledge of community services (e.g., missions and main responsibilities), the problems faced by residents in the community (e.g., inconveniences of living in community-housing), and the process and techniques for designing human-centered home devices and furniture for community residents. The learning materials of STEM knowledge and community service knowledge were developed in collaboration with industry experts, social service staff, and participating community residents. The key contents covered in STEM and community service lessons are illustrated in Table 1. Among them, the section covering techniques for designing home devices and furniture was delivered by an experienced interior designer. Videos on in-depth interviews with residents and social service staff were also presented to enable a comprehensive understanding of users' needs. A mixed-methods approach was employed to evaluate the effectiveness of the program for enhancing students' twenty-first century skills and attitudes, including (i) creative thinking, (ii) collaboration, (iii) perseverance, as well as their (iv) STEM career interests.

**Table 1:** Contents in the integrated STEM-based community service-learning course.

**Disciplines**	**Key contents**
STEM knowledge	• Coding skills and computational thinking • IoT concepts and applications • Smart home devices and examples • Maker product and its development (1 & 2)
Community service knowledge	• Community services and foundational knowledge • Community housing residents and their needs • Human-centered home devices, furniture, and the design techniques • In-depth interviews with residents and social service staff • Presentation of design solutions and collection of feedback

**Figure 2:**
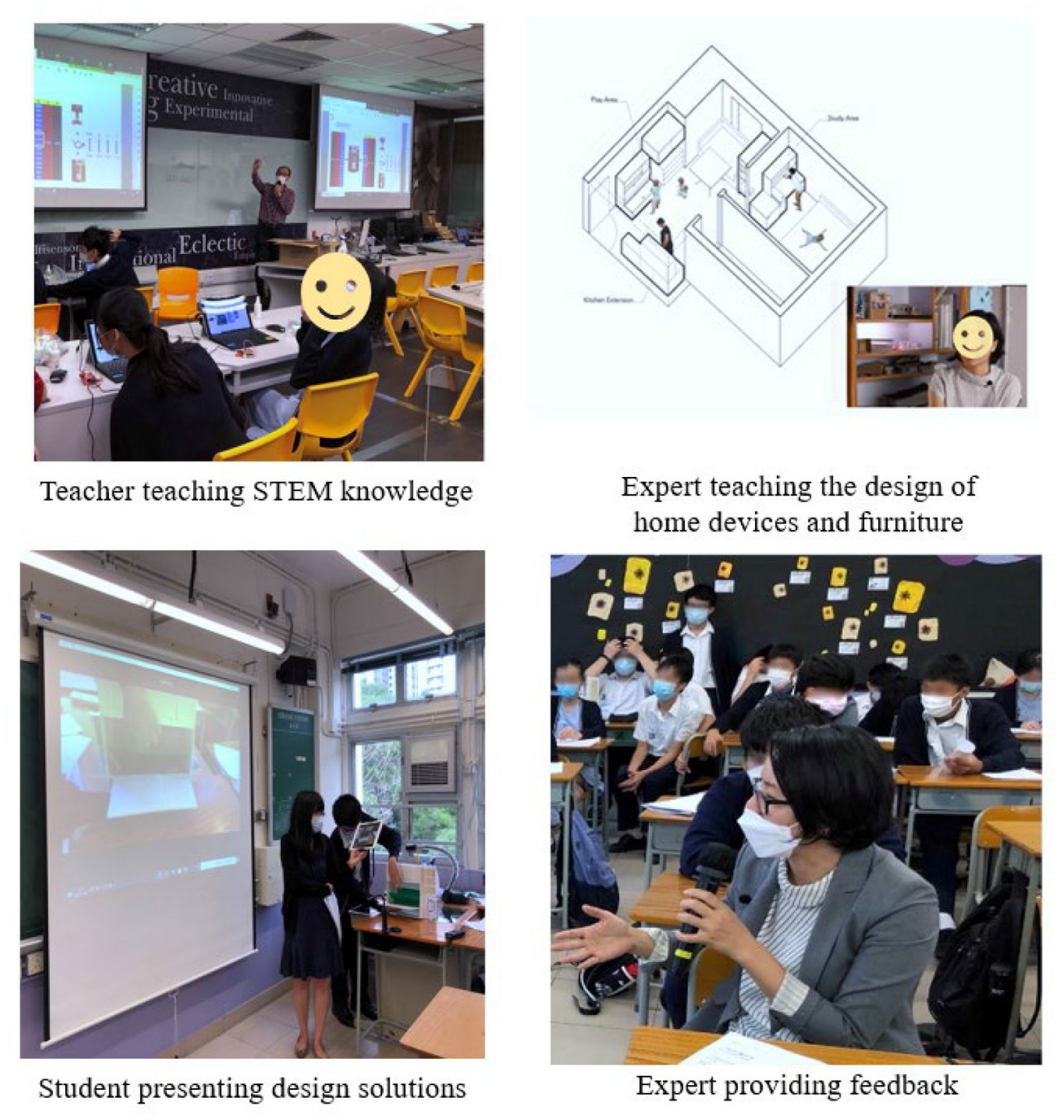
Teaching and learning activities in the program.

Students completed their initial design solutions in teams in the community service lessons. Afterwards, they presented the design solutions to experts and peers in class and solicited feedback. Next, students spent 2 weeks refining and finalizing their design solutions. Figure 2 presents some of the teaching and learning activities of the program.

### Participants and Experimental Procedures

The duration of the integrated course lasted 8 weeks, including 6 weeks for learning STEM and community service knowledge and 2 weeks for finalizing the design solution. The participants were from a junior secondary school in Hong Kong (*n* = 121). Prior to the participation in the course, students completed a pre-survey adapted from the instruments on twenty-first century skills, covering the dimensions of creative thinking, collaboration, perseverance, as well as STEM career interest (e.g., Vennix et al., 2018; Chai et al., 2020a). The same survey questionnaire was completed by the end of the program to track the changes of students in the four dimensions. Correlations between students' gender and twenty-first century skills were also explored. To triangulate with the survey results and gain more understanding of students' perceptions of the integrated course, 12 students from three groups were invited to participate in post-course interviews. The survey and interview results are reported in Section Results. Consents from students were obtained prior to participating in the research.

### Data Collection

#### Questionnaire-Based Survey

The survey questionnaire was comprised of 4 subscales and 14 items in total. The subscales were adapted from validated instruments on creative thinking (e.g., Chai et al., 2020a), collaboration (e.g., Lai and Hwang, 2014), perseverance (e.g., Toering et al., 2012), and STEM career interest (e.g., Vennix et al., 2018). The items were contextualized and translated by the authors for being adopted in the research context. To ensure the validity of the instrument, two experts in the field were invited to review and comment on the adapted items. The creative thinking subscale consisted of three items. A sample question of the creative thinking dimension is, “In STEM lessons, I can always come up with many new ideas.” The collaboration subscale consisted of three items, and a sample item is “In STEM lessons, my teammates and I usually help each other.” The perseverance subscale consisted of four items, and a sample item is “In STEM lessons, I always try my best to complete all the tasks assigned by the teachers.” The STEM career interest subscale consisted of four items, and a sample item is “I think STEM-related jobs are interesting.” Students were briefed that “in STEM lessons” refers to the lessons carried out during the integrated STEM-based community service-learning program. A six-point scale ranging from “strongly disagree” to “strongly agree” was adopted in the study. The Cronbach's alpha values for the subscales were 0.86 for creative thinking, 0.94 for perseverance, 0.94 for collaboration, and 0.94 for STEM career interest.

#### Focus-Group Interviews

Three focus-group interviews were conducted to understand students' perceptions of the integrated course. Each interview consisted of three to five students. The groups were selected from the eight project teams recommended by teachers, covering students of different engagement levels observed in the course (i.e., highly engaged and less engaged) to maximize the variety of sampling (Patton, 2001). Among the participants, there were three girls (25%) and nine boys (75%). Students were asked about how the integrated STEM course helped facilitate or nurture creative thinking, collaboration, and STEM career interest. They were also asked to articulate the challenges and opportunities they encountered as well as their recommendations for improving the program. The focus groups were audiotaped, and then transcribed for analysis. Thematic analysis was conducted to identify and report the recurring patterns of meaning (themes) (Braun and Clarke, 2006). The data was first coded by one researcher of the study, and then the second researcher randomly selected 25% of the transcripts and coded them. The percent agreement between the two researchers was 81%. Discrepancies were discussed between the two researchers until agreements were reached.

## Results

### Pre- and Post-Survey Findings

To examine whether there were any differences in students' creative thinking before and after completing the course, a paired sample *t*-test was conducted. Results indicated that there was a significant difference in the scores of post-creative thinking (*M* = 4.58, *SD* = 0.99) and pre-creative thinking (*M* = 4.07, *SD* = 1.02), *t*_(96)_ = −5.00, *p* < 0.001. See Table 2.

**Table 2:** Results of paired sample *t*-test for pre- and post-surveys.

**Dimension**	**Pre-survey**		**Post-survey**		***t-*value**	***p-*value**
	**M**	**SD**	**M**	**SD**		
Creative thinking	4.07	1.02	4.58	0.99	−5.00	<0.001
Collaboration	4.54	1.00	4.75	1.02	−2.16	0.033
Perseverance	4.41	1.07	4.77	1.00	−3.26	0.002
Career interest	3.52	1.12	4.19	1.19	−5.17	<0.001

*n = 97. Among the 121 students, 97 completed both the pre- and post-surveys*.

To examine whether there were any differences in students' collaboration before and after completing the course, a paired sample *t*-test was conducted. Results showed that there was a significant difference in the scores of post-collaboration (*M* = 4.75, *SD* = 1.02) and pre-collaboration (*M* = 4.54, *SD* = 1.00), *t*_(96)_ = −2.16, *p* = 0.033.

To examine whether there were any differences in students' perseverance before and after completing the course, a paired sample *t*-test was conducted. Results showed that there was a significant difference in the scores of post-perseverance (*M* = 4.77, *SD* = 1.00) and pre-perseverance (*M* = 4.41, *SD* = 1.07), *t*_(96)_ = −3.26, *p* = 0.002.

To examine whether there were any differences in students' career interest before and after completing the course, a paired sample *t*-test was conducted. Results indicated that there was a significant difference in the scores of post-career interest (*M* = 4.19, *SD* = 1.19) and pre-career interest (*M* = 3.52, *SD* = 1.12), *t*_(96)_ = −5.17, *p* < 0.001.

Moreover, the results revealed that career interest and creative thinking were the two most improved areas among the examined sub-dimensions.

### Correlational Findings

Correlation analyses were conducted to further understand the relationships between the subscales. The post-survey data were used in testing the correlations. As shown in Table 3, the results indicated that creative thinking, collaboration, perseverance, and career interest were significantly and highly associated, with correlations ranging from 0.550 to 0.745 (*p* < 0.01).

**Table 3:** Results of correlation test for the main variables.

**Dimension**	**1**	**2**	**3**	**4**	**5**
Creative thinking	–				
Collaboration	0.635^**^	–			
Perseverance	0.691^**^	0.745^**^	–		
Career interest	0.730^**^	0.550^**^	0.643^**^	–	
Gender	−0.125	0.033	−0.013	−0.228^*^	–

* *p < 0.05*,

** *p < 0.01. For gender, 1, male; 2, female*.

Furthermore, the results showed that gender was not significantly related to creative thinking, collaboration, or perseverance. Boys were more likely to have a higher interest in STEM careers, whereas girls were less interested. However, there were no differences regarding creative thinking, collaboration, or perseverance between girls and boys.

### Focus-Group Interviews

In the focus-group interviews, students shared their perceptions of the influences of the integrated course from three angles, including creative thinking, collaboration, and career interest. Students also shared the difficulties they encountered and how they coped with them. In general, students appreciated the positive changes brought by the program. Students also made several suggestions for further improving the program.

Regarding creative thinking, most students expressed that their creative thinking skills improved after completing the program. Several students stated that the program pushed them to think deeper and more thoroughly, and helped them to be more productive in generating new ideas (Gralewski and Karwowski, 2019). For example, one student commented that: “*This project boosted our creativity. I was not so creative in daily life. But, when I worked on this project, I threw up many new ideas*.” They also expressed that they would look at different angles to ensure the idea generated is not just “novel” but also “appropriate” (Runco et al., 2005). For example, one student elaborated that: “*In the design process, we would think deeper and consider more factors. In the past, we would not have thought about so many aspects. Now, we would consider whether there could be any errors if we put it into production, and whether existing technology is ready for implementing our design ideas, etc*.”

Moreover, students described that they used adaptation and synthesis skills to improve the design solutions, which are considered important indicators of creativity (Ramalingam et al., 2020). For example, one student shared that “*In one session, all teams presented their design solutions, from which we learned about the weaknesses of our designs and others'. So, we synthesized what we learnt from this experience, and enhanced our final design solution*.”

As for the aspect of collaboration skills, students expressed that their collaboration skills were enhanced through this learning experience. For example, one student commented, “*I felt that my collaboration skills become better, because we often work together to formulate the design concept, as well as the physical structure of the product*.” The experience also improved students' eagerness to communicate and strengthened their closeness. For example, another student manifested that: “*At first, I did not like to share my viewpoints, but after participating in more group discussions, I became more willing to communicate with teammates. The collaboration and communication further enhanced the closeness between our teammates*.”

Students also expressed that they were actively adapting their approach in collaboration. One student expressed that: “*I used to spend a long time conceptualizing an idea, and would only share it when it was fully developed. Now, (to be more effective) I would share all my initial ideas, and work with my peers to find the best solution*.” In fact, the willingness to work with others and share ideas with peers is considered important enabler of creative thinking (Foster and Schleicher, 2022). The positive feedback on collaboration, in a sense, explained why students had higher creative thinking after completing the program.

Regarding perseverance, we wanted to find out whether students had encountered difficulties in their studies and whether they exerted continuous efforts to resolve them, i.e., an indicator of perseverance (Toering et al., 2012). Hence, we raised the following questions, “Did you meet any difficulties in completing the project, and how did you cope with them?” Most of the interviewees commented that they did not encounter many difficulties in completing the project because they functioned as a team. One student shared that they met technical problems in designing their solution, and they solved them by searching the website and reading relevant technical posts. Another student mentioned that it took them a long time to record an introduction video on the design solution. Though it was quite challenging and time-consuming, they insisted on discussing and clarifying the details of each scene and provided timely advice to the students in charge of filming. Another challenge reported by students was the time restriction. Another challenge reported by the students was the time constraint. One student repeatedly mentioned that though they had 2 weeks to refine and finalize their design solution, he felt that the time was still a bit tight and hoped that more time was assigned to refine the solutions. Although this point is not directly related to perseverance, it reminds us of what to pay attention to when designing future projects. We can allow more time to incubate, reflect and select among alternative design solutions (Sternberg, 2003).

During the interview, we found that girls were more interested in STEM subjects and felt more at ease with coding activities than they were before participating in the program, but they did not yet have a plan for their future careers. Meanwhile, the program has motivated some boys to consider pursuing STEM as their future career. One boy expressed that he was thinking about whether to work in STEM-related fields after graduation but had not decided yet. Another boy said that though he would like to work in other fields in the future, he would discover more about STEM and pursue it as a hobby. The results indicated that the program positively impacted students' interest in STEM and related careers, but further efforts are needed to foster a stronger interest in the profession.

## Discussion

This study proposed an integrated STEM-based community service-learning model referring to the componential model of creativity and the design thinking model. The model advocates the engagement of students in design challenges to solve real-world problems faced by community residents, while providing students with access to learning domain knowledge in STEM and community service. Moreover, it encourages the establishment of a supportive learning environment for students by offering expert or user feedback. Other resources and support from the school level can also be provided depending on the context of each study. In our program, to ensure the authenticity of the learning materials, the program team collaborated with industry experts, staff of social service organizations, and community residents. The designing home devices and furniture section was delivered by an industry expert in video format. The survey results showed that students increased their creative thinking, collaboration, perseverance, and STEM career interest, especially in creative thinking and career interest, upon completion of the program.

The interview findings are consistent with the survey results. The positive results support the assumption that when students are provided with adequate resources, domain-specific skills, and a well-structured creative design process, twenty-first century skills can be improved. The exploration of solving community service problems also benefits local community residents (Andreoletti and Howard, 2018). In the future, we can promote the model in more K-12 schools and examine its effectiveness.

As Amabile and Pratt (2016) stated, the incubation of creative thinking and innovation requires three essential elements, such as resources, domain-specific skills and process skills, and a stimulating and supportive learning environment. In this program, we developed an integrated course collaborating with industry experts, social service staff, and community residents to equip students with domain-specific knowledge and foundations for designing user-centered products. We also guided students' design in a step-by-step manner. Furthermore, to better support students, we created an opportunity to interact with industry experts and social service staff to collect feedback on the feasibility of their design solutions. These practices promoted an improvement in creative thinking. The result is consistent with the finding of Nazzal and Kaufman's (2020) work that creative thinking is connected to domain-specific knowledge. In addition, the design challenge was carried out in the form of teamwork, and the positive challenge and collaboration in teams may have encouraged creative thinking (Maiorca et al., 2021). Moreover, the authentic problem-based learning process may have sparked students' interest in STEM subjects and related careers, and consequently, promoted their creativity (Bush et al., 2020).

Perseverance drives people to achieve their goals despite obstacles and difficulties (Pury, 2009). This integrated course promoted students' perseverance, supporting the previous finding that the authenticity of learning tasks and materials contributes to higher perseverance in learning (Mutlu and Yildirim, 2019). In addition, our result on the positive correlation between perseverance and creative thinking is consistent with Scherer and Gustafsson's (2015) finding that perseverance is positively correlated with creative problem-solving.

Collaboration is a predictor of highly effective problem-solving teams (Fullan and Langworthy, 2014). Chai et al. (2020a) examined the core dimensions of twenty-first century skills, and confirmed that collaborative learning predicts students' creative problem-solving. Hsia et al. (2021) found that promoting collaborative learning and interaction in class could enhance students' creative thinking tendency. Our correlation test result matches their findings. In the future, we can continue to encourage collaboration among students and explore the connections between group styles, creative thinking, and STEM career interest.

Our survey results indicated that students' STEM career interest increased from a low level (3.5) to a relatively higher level (4.19), which corroborates the survey finding of Vennix et al. (2018). The survey results showed that STEM courses are more likely to have a positive impact on STEM career interest when they are linked to real-life contexts. The interview results are consistent with the survey results. Most students expressed that their interest in STEM is higher than before. In addition, although gender was not correlated with creative thinking in STEM, gender was weakly correlated with career interest (i.e., girls had relatively lower career interest). The result suggests that girls are less likely to choose STEM careers in the future, despite their potential to play an equally important role in STEM fields (Ünlü and Dökme, 2020). Further work on how to promote female students' career interest in STEM is worth additional research efforts.

## Implications, Limitations, and Future Research

This study has important theoretical and practical implications for science education. From a theoretical perspective, we proposed an integrated STEM-based community service learning model that builds on the strengths of the componential theory of creativity and the EDIPT design thinking model, one of the earliest theoretical attempts in the cross-disciplinary field of STEM education and community service. Integrating STEM with community service learning is a nascent field, and thus there is a paucity of empirical research and theoretical exploration in this area (Collins et al., 2020). This model analyzes the core elements (e.g., resources, domain knowledge, and process) that enable individual or team innovation, and illustrates the procedure for organizing the design activities. Furthermore, the model links real-life community service issues, community service learning, STEM education, and design activities, and empowers cross-disciplinary learning with meaning (i.e., solve authentic problems to improve the comfortable and convenience of community residents' lives).

From the perspective of practice, the study demonstrated the process for integrating community service learning and STEM education following the integrated model. The study also exhibited how contents can be developed building upon the multiple expertise of industry experts, teachers, and social service staff. Moreover, the pre- and post-implementation results showcased the potential outcomes that could be achieved through this sort of integrated attempt regarding students' twenty-first century skills, perseverance, and STEM career interest.

Practitioners in science education and humanities can also refer to this model for designing interdisciplinary curriculum to nurture creative thinking while strengthening attitudes, values, and sense of social responsibility. Teachers are always important stakeholders of any new educational initiatives, playing a salient role in the adoption process (Geng et al., 2019; Jong, 2019); there should be no exception in STEM education (Chai et al., 2019). Training in understanding and implementing this interdisciplinary curriculum can be organized for teachers to remove barriers to adopting new pedagogical approaches, facilitate their smooth implementation of this innovative teaching model, and ultimately improve student performance (Jong et al., 2021; Lau and Jong, 2022).

Despite its strengths, this study also has some limitations. First, a limitation of the study is that it mainly used a one-group pre- and post-survey approach to understand the impact of this integrated course. Future studies that include a control group would produce more rigorous results. In addition, we encourage future studies to collect different data sets, such as tests or artifacts to further understand the impacts of the STEM-based community service-learning model. Second, given that the program was carried out in a secondary school in Hong Kong, its generalizability to other settings needs to be tested. In the future, this integrated learning approach can be extended to more schools in different settings and see whether consistent results could be achieved. Moreover, this study mainly explored the combination of STEM with the theme of community service learning such as designing home devices and furniture for residents in community-housing. Future studies can also explore the applicability of other community service themes.

## Conclusion

This study provides quantitative evidence that integrating STEM with community service learning is beneficial to promoting students' creative thinking, collaboration, perseverance, and STEM career interest. The qualitative interviews with the students further validated these quantitative findings. Scardamalia et al. (2012) advocated that citizen in the twenty-first century need to add value to existing knowledge by creating new knowledge or designing new artifacts that are valuable to others. Our STEM-based community service program is a small step toward that ideal and provides a concrete example of how this could be done in the educational context.

## Data Availability Statement

The datasets presented in this article are not readily available because the access of the datasets should be approved by the involved schools. Requests to access the datasets should be directed to Morris Siu-Yung Jong, mjong@cuhk.edu.hk.

## Ethics Statement

The studies involving human participants were reviewed and approved by the Chinese University of Hong Kong. The participants/their legal guardians provided the informed consent to participate in this study.

## Author Contributions

BH: conceptualization, visualization, data collection, data analysis, writing the first draft of the manuscript, and revision. MS-YJ: conceptualization, visualization, funding acquisition, project administration, supervision, validation, rewriting sections of the manuscript, review, and edit. RK: rewriting sections of the draft, visualization, review, and edit. C-SC and MY-CJ: visualization, validation, review, and edit the manuscript. All authors approved the submission.

## Funding

The work described in this paper was supported by the Hong Kong Jockey Club Charities Trust (Project Title: Jockey Club Community Care and STEM in Action [Project S/N Ref: JC 2019/0112]).

## Conflict of Interest

The authors declare that the research was conducted in the absence of any commercial or financial relationships that could be construed as a potential conflict of interest.

## Publisher's Note

All claims expressed in this article are solely those of the authors and do not necessarily represent those of their affiliated organizations, or those of the publisher, the editors and the reviewers. Any product that may be evaluated in this article, or claim that may be made by its manufacturer, is not guaranteed or endorsed by the publisher.
